# Supplementary material for: Evaluation of Different Natural Ventilation Strategies by Monitoring the Indoor Air Quality Using CO2 Sensors
Source: Int J Environ Res Public Health. 2023 Sep 14;20(18):6757. doi: 10.3390/ijerph20186757 (PMC10531387; doi:10.3390/ijerph20186757)
Supplement: Supplementary file 1 [file ijerph-20-06757-s001.zip › ijerph-2519799-supplementary.pdf]

International Journal of
Environmental Research and Public Health

MDPI

Supplementary Materials:
Table S1. Additional classroom information

School	Students age (years old)	Nº of students	Classroom volume (m³)	Volume ratio (m3/st.)	Volume per total body unit (m3/kg)	ARIAM sensor used	Sensor location (m from floor)	Measuring timeframe	Pressure (hPa)	Location	Weather conditions	Other relevant conditions
CEIP Ciudad de Bolonia	9-10	19	157.7	8.3	0.27	1	1	9:00-13:30	-	Urban school near green areas	Sunny day with 20ºC average. No wind (0,3 m/s average velocity).	Heaters off (in the wall). Shutters with drawers. Disorder in the class.
CEIP Jaume I	9-10	20	171.5	8.56	0.28	1	1	9:00-13:30	-	Urban school with traffic surrounding but next to a green area	Windy day. Gusts of wind up to 14,2 m/s.	Heaters on (in the wall). No shutters, only curtains. 4 big windows and windows to the corridor.
CEIP 8 de Març	10-11	22	184.3	8.38	0.25	1	1	9:00-10:00 and 11:30-13:00	-	Urban school near green areas	Sunny day with 15,2 ºC average temperature. The average windy velocity of 0,5 m/s.	Heater on. Shutters. Two windows with two sashes and one fix part each. Two ceiling ventilators. Projector
	11-12	22	311	14.14	0.39	1	1	10:00-11:00				Music classroom. Heater. Seven windows. Previously to the measurements, there was another session with 22 students
CEIP Humanista Mariner	5	21	60.8	2.89	0.16	1	1	9:00 - 11:00	-	Urban school near green areas	Windy with an average windy velocity of 4,2 m/s and wind's guts up to 13,3 m/s.	Heater on.
	4	21	60.8	2.89	0.17	1	1	11:40 - 12:40	-			
	11	27	142.1	5.26	0.16	1	1	15:00 - 17:00	-			
	9-10	15	197.1	13.14	0.42	2	1	8:50 - 11:34	955.05		Sunny day with an	NW orientation, which faced the mines.
	8-9	17	197.1	11.59	0.43	1	1	11:45-13:30	953.1			

CEIP Fabián y Fuero										Rural school near a open mine	average temperature of 14,1 ºC and an average wind velocity of 2,2 m/s (10,2 m/s maximum wind velocity).	Shutters down due to the sun and desks with dust. Heater on.
	8-9	16	180.9	11.31	0.42	2	1	15:34-17:30	953.05			Laboratory. Same conditions
CEIP San Juan de la Ribera	9-10	15	105.4	7.02	0.23	1	1	9:30-11:30	1015.42	Urban school in the city centre with no traffic	Sunny day with an average temperature of 13 ºC and wind of 1,1 m/s (max. wind velocity ten m/s).	The heater was on at 7:54h. Cleaning of the windows during the break. Good isolation.
	8-9	24	123.1	5.13	0.19	1	1	11:30-14:00	1015.42			
EI Febes	1-2	12	107.6	8.97	0.90	1	0.8	9:00-13:15 and 15:00-16:15	1018.2	Urban school near green areas	The average temperature of 12 ºC and an average windy velocity of 1,1 m/s (max. wind velocity 5,5 m/s).	Forced extraction for 15 minutes at 10:15h and 11:30h. Air conditioning at 25 ºC. Anti-finger doors
IES La Serranía	15-16	23	148.4	6.45	0.14	1	1	8:50-13:51	965.41	Rural school near a open mine	The average temperature of 12,6 ºC and an average windy velocity of 0,3 m/s (max. wind velocity 4,2 m/s).	When arriving at the class, everything was closed, and the CO_2 values were already higher than the outside. Good isolation.

School	Students age (years old)	Nº of students	Classroom volume (m³)	Volume ratio (m3/st.)	Sensor location (m from floor)	Measuring timeframe	Pressure (hPa)	Weather conditions	Other relevant conditions
CEIP Ciudad de Bolonia	9-10	19	157.7	8.3	1	9:00-13:30	-	Sunny day with 20ºC average. No wind (0,3 m/s average velocity).	Heaters off (in the wall). Shutters with drawers. Disorder in the class.

CEIP Jaume I	9-10	20	171.5	8.56	1	9:00-13:30	-	Windy day. Gusts of wind up to 14,2 m/s.	Heaters on (in the wall). No shutters, only curtains. 4 big windows and windows to the corridor.
CEIP 8 de Març	10-11	22	184.3	8.38	1	9:00-10:00 and 11:30-13:00	-	Sunny day with 15,2 ºC average temperature. The average windy velocity of 0,5 m/s.	Heater on. Shutters. Two windows with two sashes and one fix part each. Two ceiling ventilators. Projector
	11-12	22	311	14.14	1	10:00-11:00			Music classroom. Heater. Seven windows. Previously to the measurements, there was another session with 22 students
CEIP Humanista Mariner	5	21	60.8	2.89	1	9:00 - 11:00	-	Windy with an average windy velocity of 4,2 m/s and wind's guts up to 13,3 m/s.	Heater on.
	4	21	60.8	2.89	1	11:40 - 12:40	-		
	11	27	142.1	5.26	1	15:00 - 17:00	-		
CEIP Fabián y Fuero	9-10	15	197.1	13.14	1	8:50 - 11:34	955.05	Sunny day with an average temperature of 14,1 ºC and an average wind velocity of 2,2 m/s (10,2 m/s maximum wind velocity).	NW orientation, which faced the mines. Shutters down due to the sun and desks with dust. Heater on. The 180,9 m^3 classroom is a laboratory.
	8-9	17	197.1	11.59	1	11:45-13:30	953.1		
	8-9	16	180.9	11.31	1	15:34-17:30	953.05		
CEIP SJR	9-10	15	105.4	7.02	1	9:30-11:30	1015.42	Sunny day with an average temperature of 13 ºC and wind of 1,1 m/s (max. wind velocity ten m/s).	The heater was on at 7:54h. Cleaning of the windows during the break. Good isolation.
	8-9	24	123.1	5.13	1	11:30-14:00	1015.42		
EI Febes	1-2	12	107.6	8.97	0.8	9:00-13:15 and 15:00-16:15	1018.2	The average temperature of 12 ºC and an average windy velocity of 1,1 m/s (max. wind velocity 5,5 m/s).	Forced extraction for 15 minutes at 10:15h and 11:30h. Air conditioning at 25 ºC. Anti-finger doors
IES La Serranía	15-16	23	148.4	6.45	1	8:50-13:51	965.41	The average temperature of 12,6 ºC and an average windy velocity of 0,3 m/s (max. wind velocity 4,2 m/s).	When arriving at the class, everything was closed, and the CO_2 values were already higher than the outside. Good isolation.

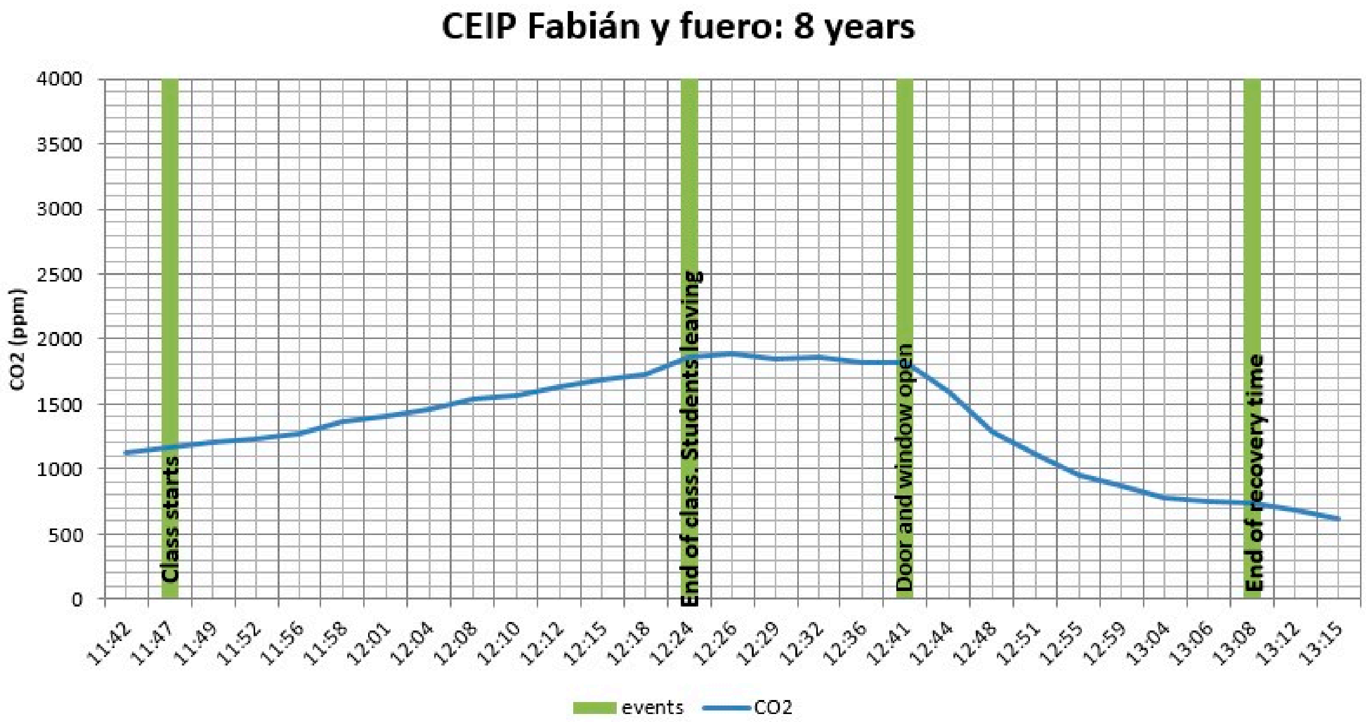

Figure S1. CO_2 - events in CEIP Fabian y Fuero 8 years.

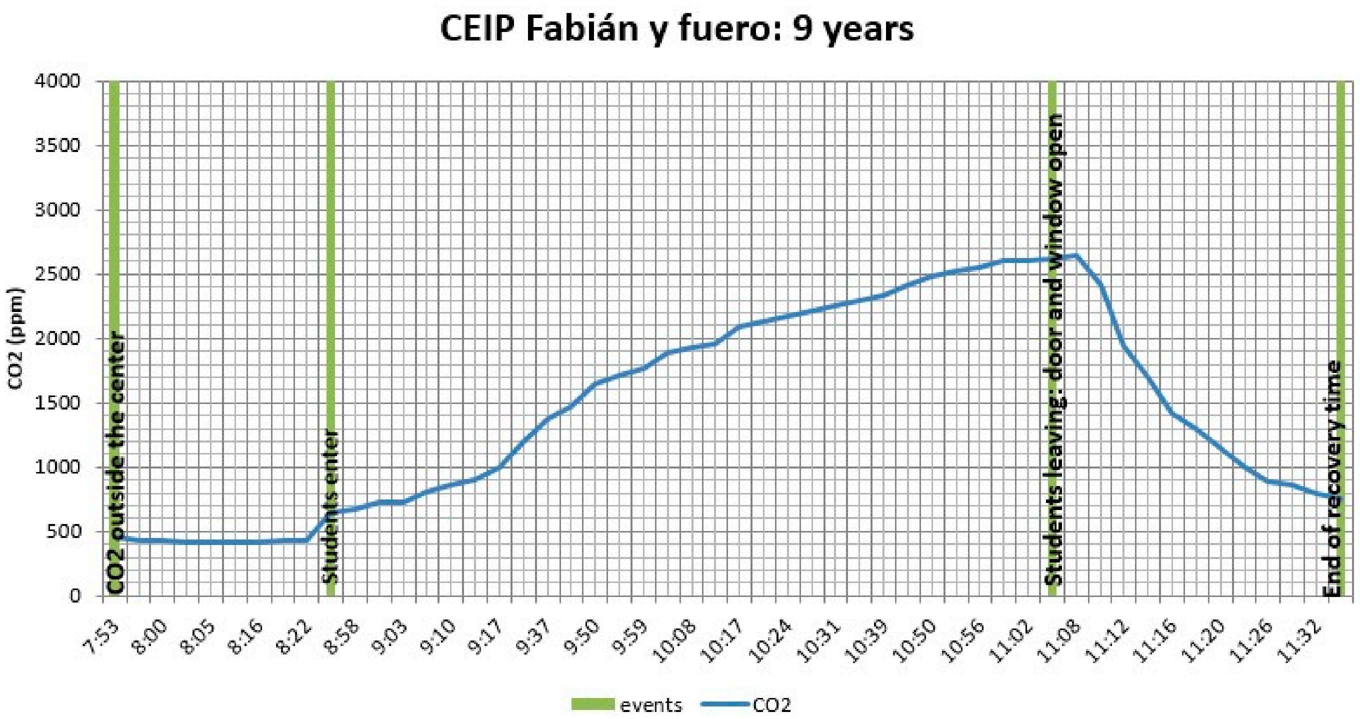

Figure S2. CO_2 - events in CEIP Fabian y Fuero 9 years.

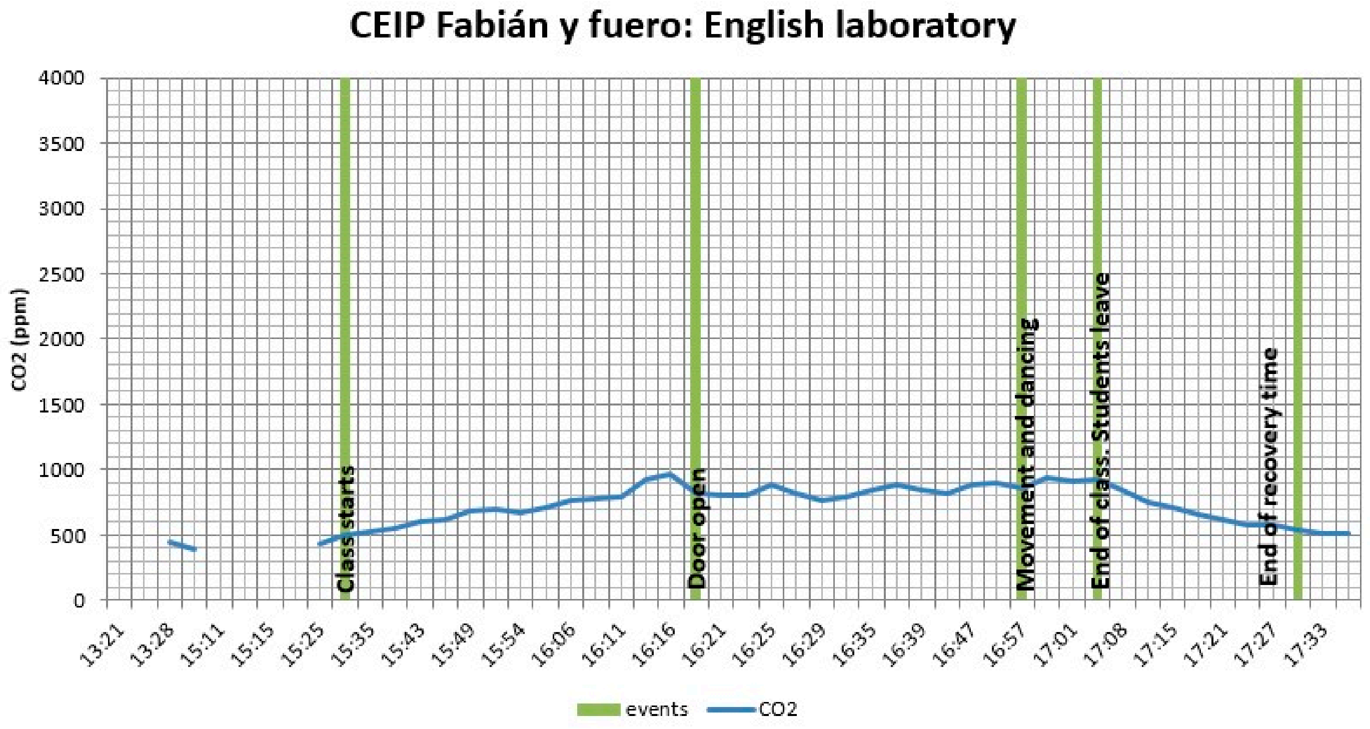

Figure S3. CO_2 - events in CEIP Fabian y Fuero English lab

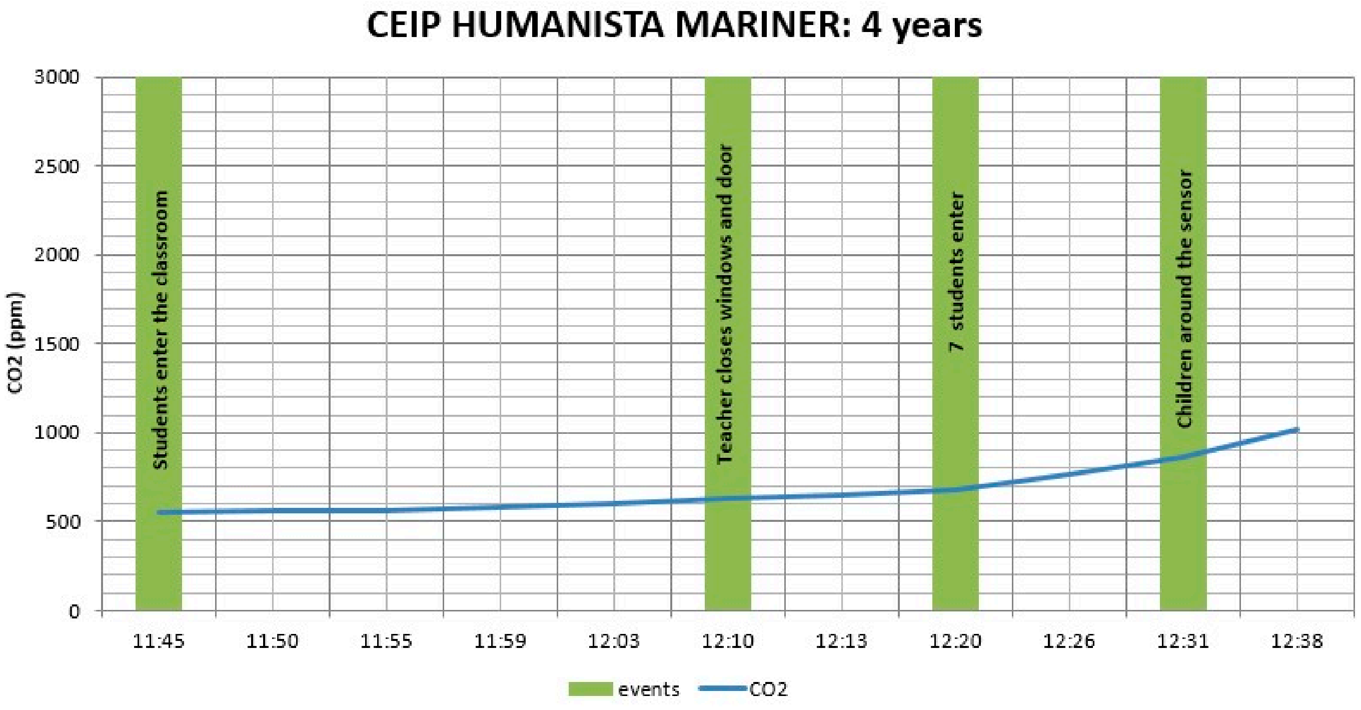

Figure S4. CO_2 - events in CEIP Humanista Mariner 4 years

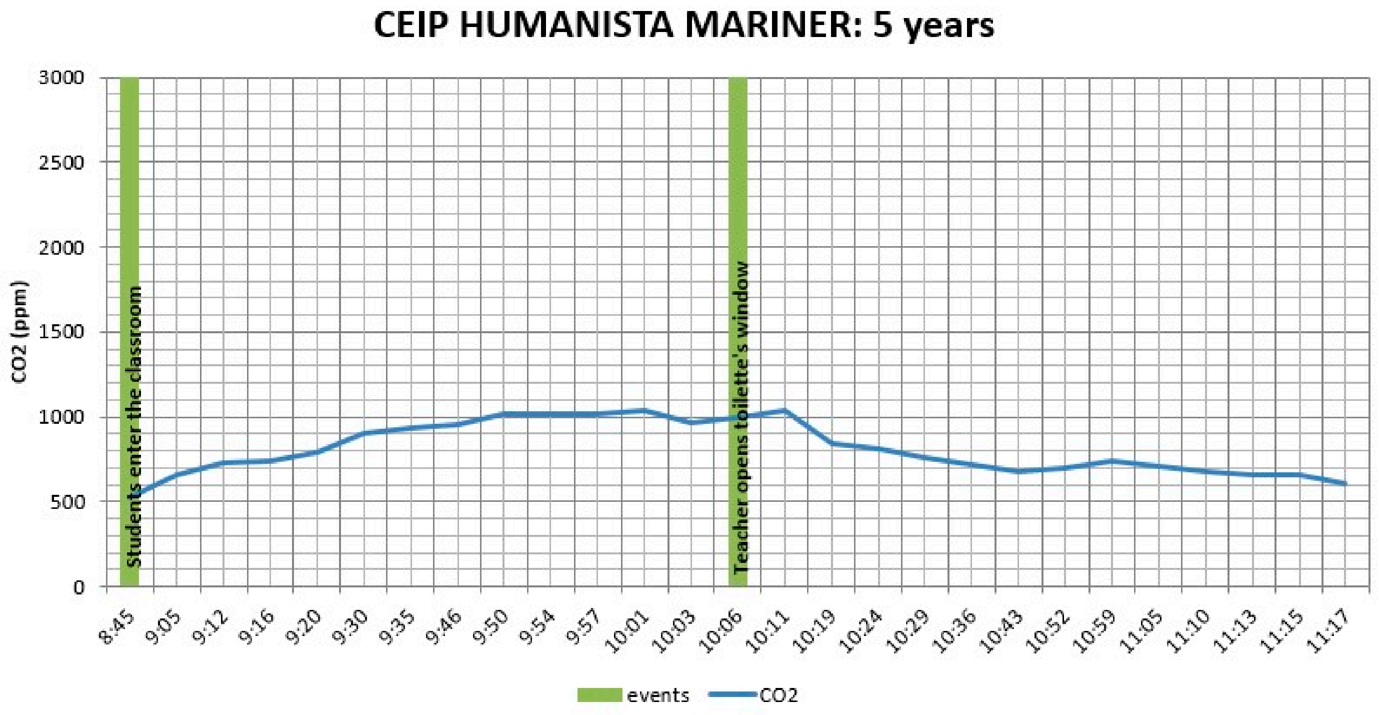

Figure S5. CO_2 - events in CEIP Humanista Mariner 5 years

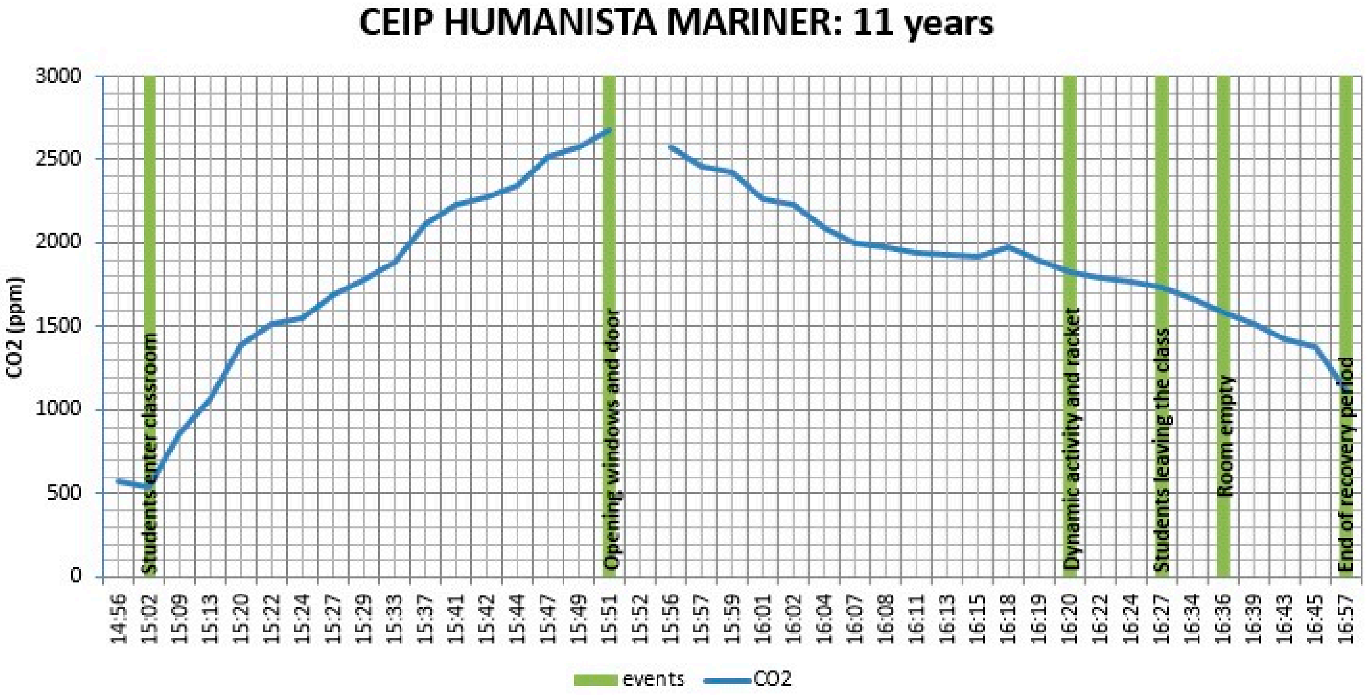

Figure S6. CO_2 - events in CEIP Humanista Mariner 11 years

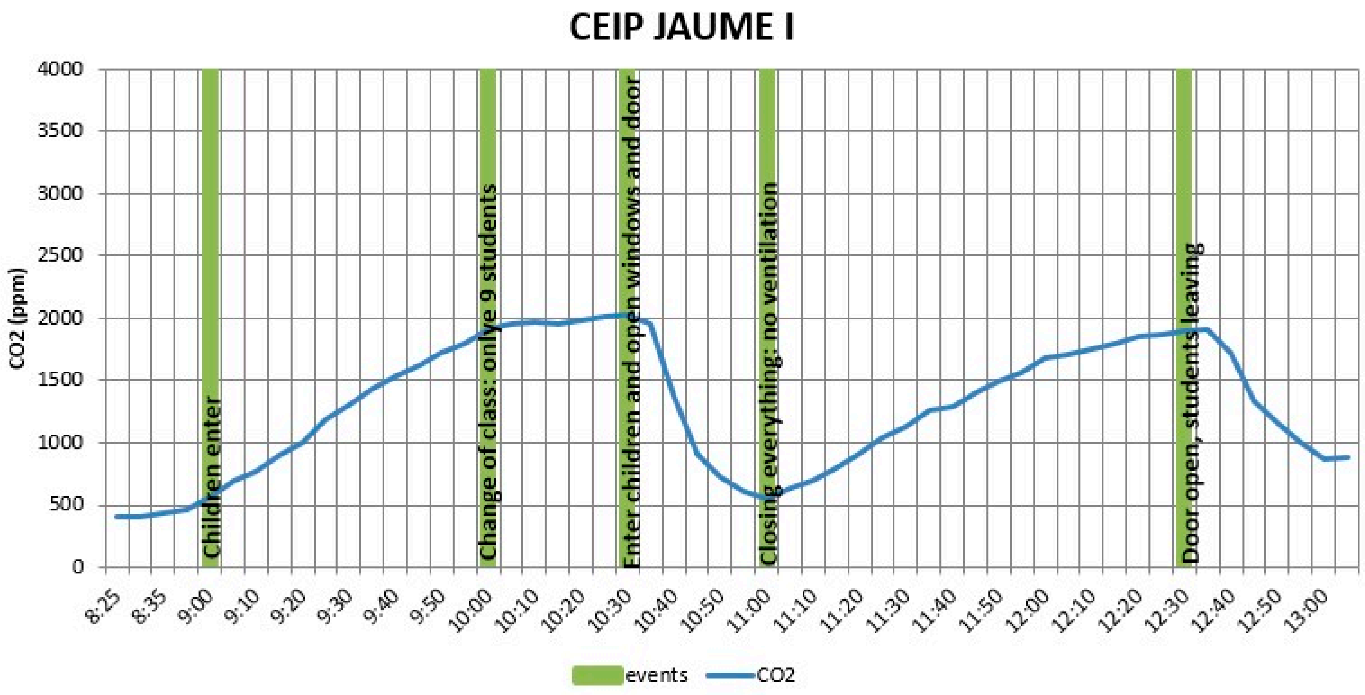

Figure S7. CO_2 - events in CEIP Jaume I

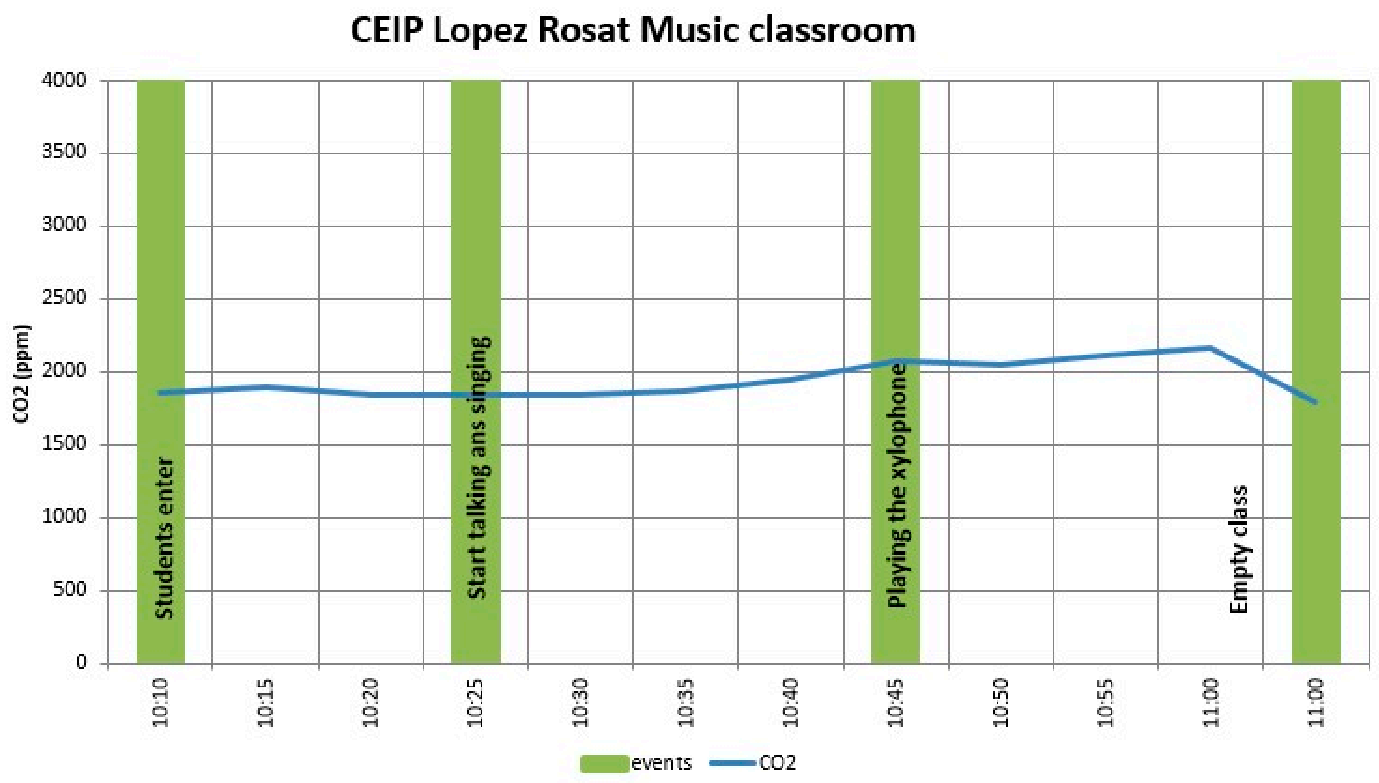

Figure S8. CO_2 - events in CEIP López Rosat Music classroom

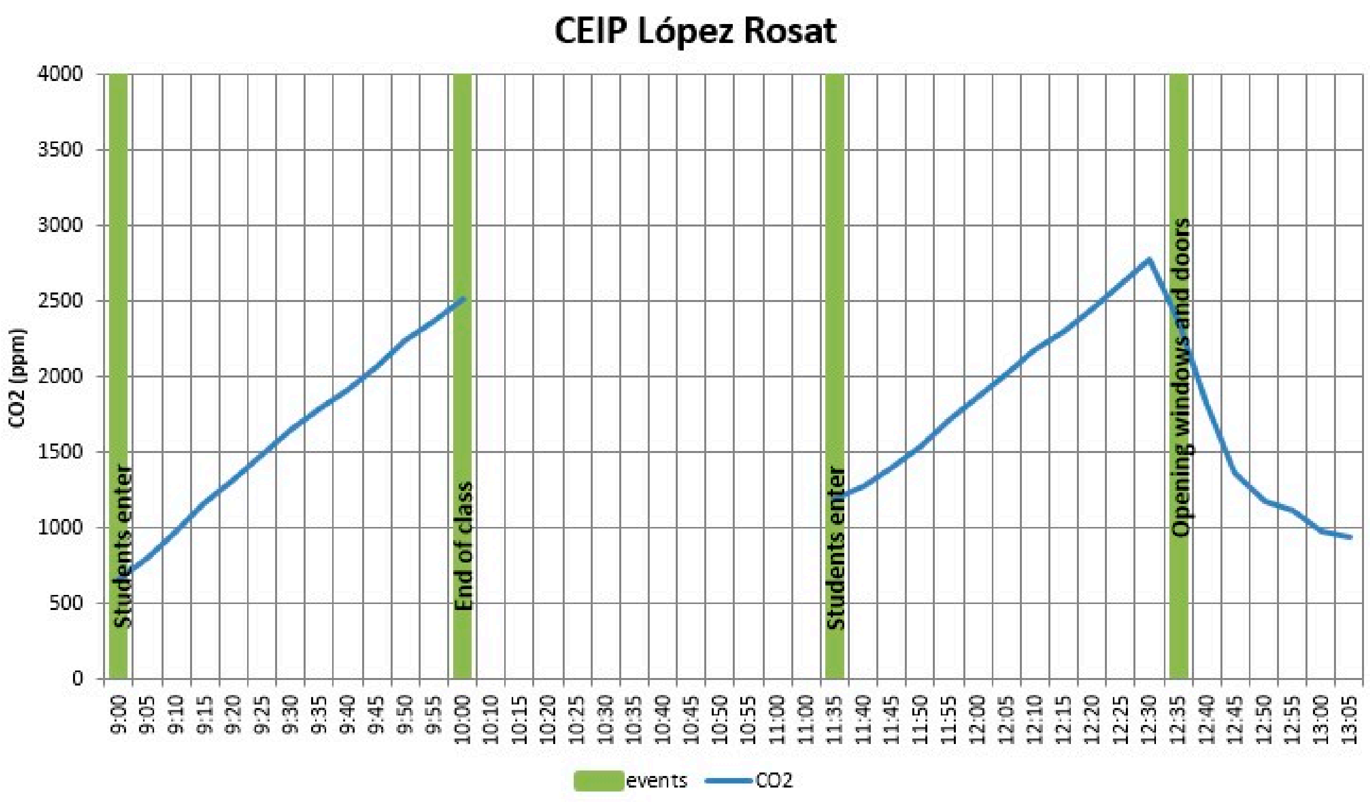

Figure S9. CO_2 - events in CEIP 8 de Març

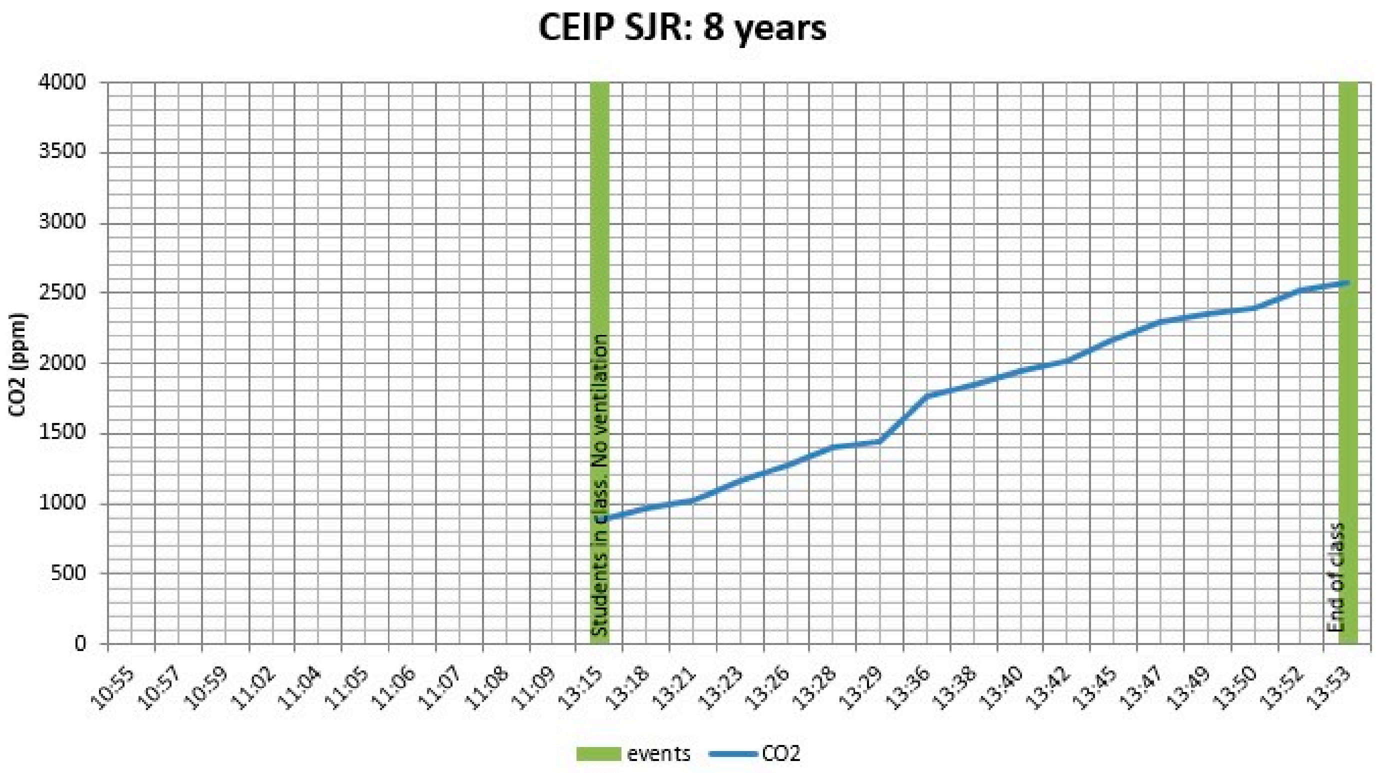

Figure S10. CO_2 - events in CEIP SJR 8 years

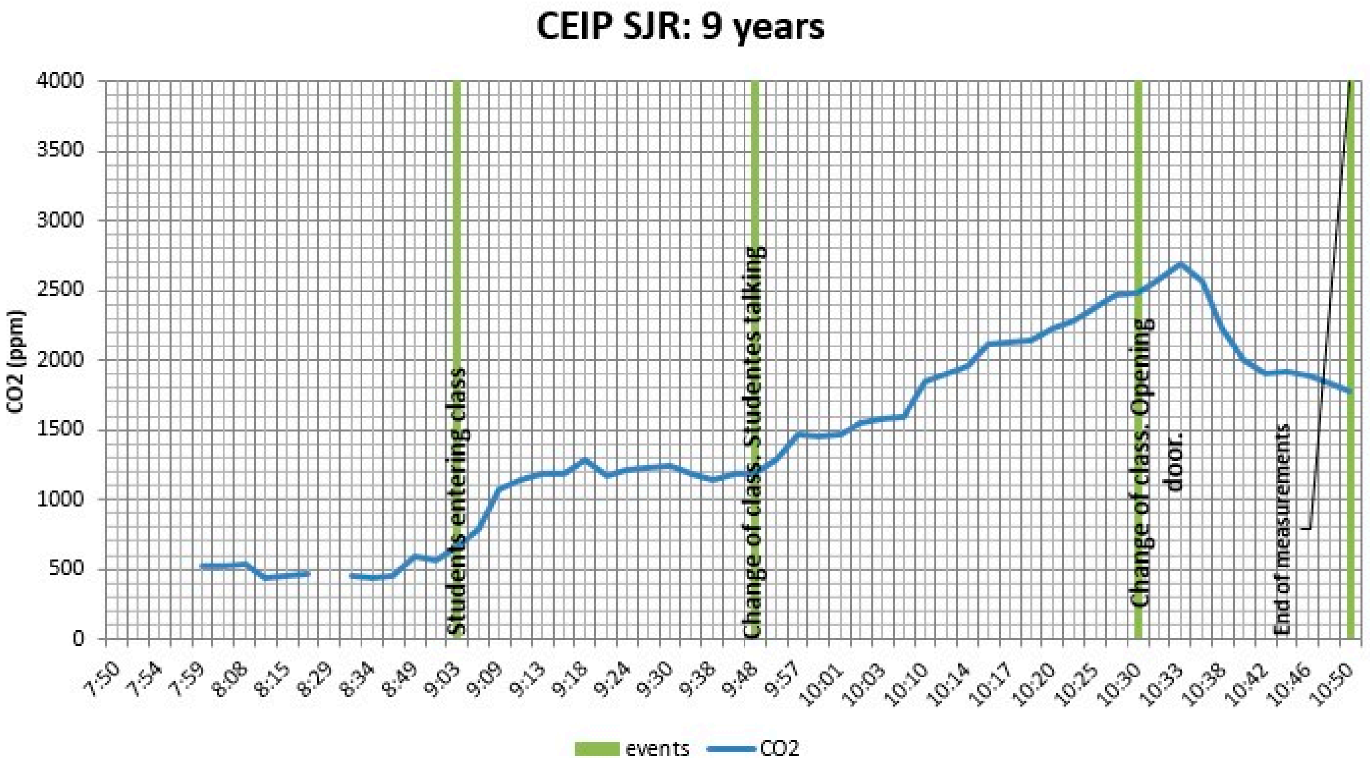

Figure S11. CO_2 - events in CEIP SJR 9 years

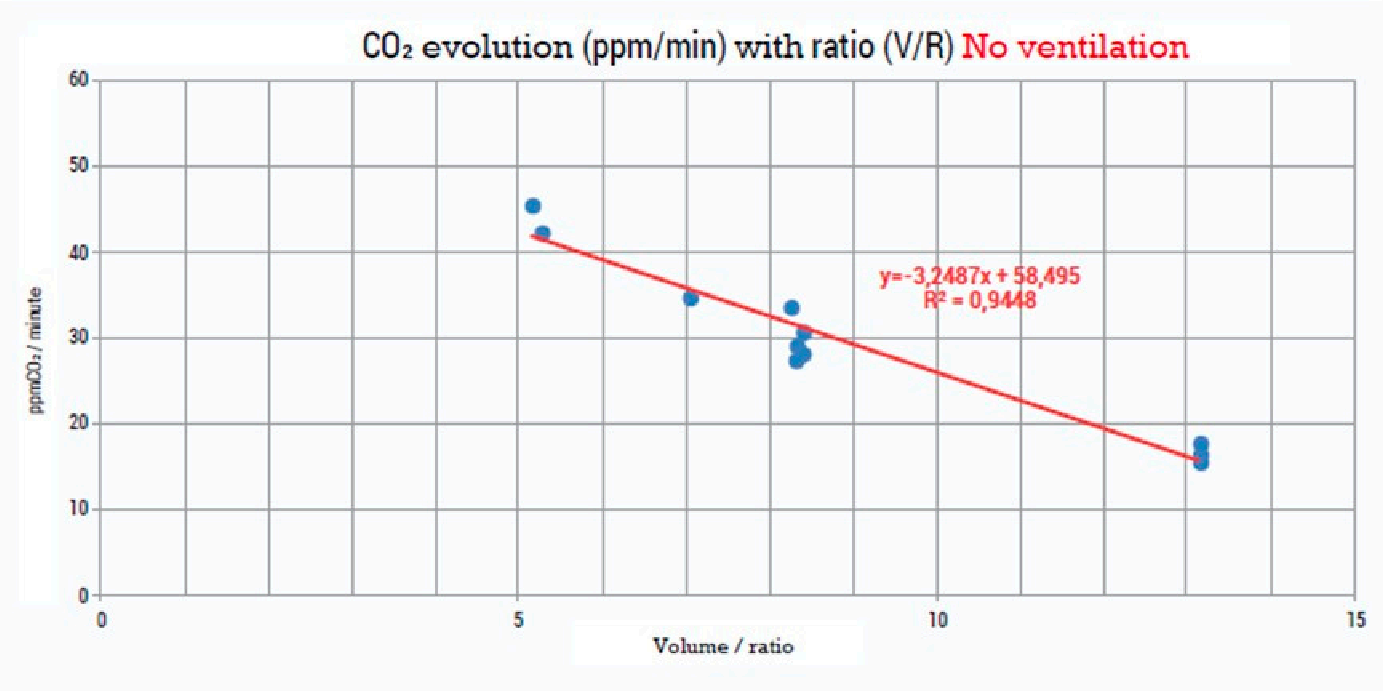

Figure S12. Correlation between CO_2 evolution and volume ratio
